# Native holdup (nHU) to measure binding affinities from cell extracts

**DOI:** 10.1126/sciadv.ade3828

**Published:** 2022-12-21

**Authors:** Boglarka Zambo, Bastien Morlet, Luc Negroni, Gilles Trave, Gergo Gogl

**Affiliations:** ^1^ Institut de Génétique et de Biologie Moléculaire et Cellulaire (IGBMC), INSERM U1258/CNRS UMR 7104/Université de Strasbourg, 1 rue Laurent Fries, BP 10142, Illkirch F-67404, France.; ^2^ Équipe Labellisée Ligue 2015, Département de Biologie Structurale Intégrative, Institut de Génétique et de Biologie Moléculaire et Cellulaire (IGBMC), INSERM U1258/CNRS UMR 7104/Université de Strasbourg, 1 rue Laurent Fries, BP 10142, Illkirch F-67404, France.

## Abstract

Characterizing macromolecular interactions is essential for understanding cellular processes, yet most methods currently used to detect protein interactions from cells are qualitative. Here, we introduce the native holdup (nHU) approach to estimate equilibrium binding constants of protein interactions directly from cell extracts. Compared to other pull-down–based assays, nHU requires less sample preparation and can be coupled to any analytical methods as readouts, such as Western blotting or mass spectrometry. We use nHU to explore interactions of SNX27, a cargo adaptor of the retromer complex and find good agreement between in vitro affinities and those measured directly from cell extracts using nHU. We discuss the strengths and limitations of nHU and provide simple protocols that can be implemented in most laboratories.

## Teaser

Native holdup can provide estimations for thousands of equilibrium binding constants directly from cell extracts.

## INTRODUCTION

Quantitative high-throughput (HTP) approaches are needed to explore the human protein-protein affinity interactome (*1*–*3*)⁠. Commonly, protein complexes are identified from cellular extracts with pull-down–based approaches involving a bait immobilized on beads via antibodies (immunoprecipitation) or affinity tags (affinity purification). Protein complexes are then purified from cell extracts on the beads by applying extensive washing protocols that reduce nonspecific background stepwise while retaining the bound specific interaction partners (*4*, *5*). Other methods used for HTP discovery of protein complexes are based on two-hybrid screens, the cofractionation of molecular assemblies, display technologies, or the irreversible modification of proteins found in close proximity, and are often coupled to a pull-down–based approach (*6*–*10*)⁠. All these methods are powerful, yet they remain qualitative and do not shed light on the biophysical attributes underpinning the observed interactions, such as dissociation constants (*K*_d_).

Molecules neither “interact” nor “not interact.” Rather, their interactions follow physical rules. For example, the law of mass action in binding equilibrium defines the degree of complex formation as a function of binding affinities and cellular concentrations. Quantitative assays aiming to measure binding affinities at HTP are mostly limited to fragmentomic approaches, where interactions are studied between minimal binding fragments, most usually between globular domains and peptide motifs. Most recently, we characterized tens of thousands of domain-motif affinities using the holdup assay, a single-point binding experiment that measures the degree of complex formation under equilibrium (*11*, *12*)⁠. These and other recent advances brought proteome-wide fragmentomic affinity screening of elementary reactions within reach (*13*–*16*)⁠. However, fragmentomic approaches do not reveal how affinities change when minimal binding fragments are embedded in the full-length proteins or even larger macromolecular complexes.

As a solution, we developed the quantitative native holdup (nHU) assay to estimate binding affinities of full-length proteins directly from native cell extracts. We demonstrate that nHU can be coupled to various protein analytical methods, such as Western blot (WB) or mass spectrometry (MS), exploiting all advances of protein analytics. We explore the interactions of Sorting Nexin 27 (SNX27), a component of the retromer complex involved in the endosome-to-plasma membrane protein recycling (*17*, *18*)⁠. We find that the nHU assay provides robust estimations about apparent equilibrium binding constants over a wide affinity range. We show that nHU and fragmentomics are highly complementary as affinities measured with these approaches are related but not necessarily identical since apparent binding properties of full-length proteins can be modulated by many phenomena, such as multivalency or conformational heterogeneity.

## RESULTS

### Principles of nHU

In a holdup experiment, an analyte solution is incubated with either control or bait-saturated resin, and the differential depletion of prey protein within the liquid phase fraction is measured with analytical methods to calculate the equilibrium binding constant (*11*, *12*, *19*)⁠. We used streptavidin resin saturated with biotinylated peptides or proteins as baits and biotin or biotinylated-maltose-binding protein (MBP) as controls. The saturated resin is equilibrated with the analyte, and the liquid phase is rapidly separated from the resin either by fast filtration or by pipetting the supernatant after a brief centrifugation. The unbound prey fraction is quantified by the relative prey concentration between bait and control samples, and the complementary bound prey fraction, conventionally called binding intensity (BI), is determined as follows

$$\mathrm{BI}=1-\frac{C_{\text{free prey}\;(\text{measured in bait experiment})}}{C_{\text{total prey}\;(\text{measured in control experiment})}}$$ (1)

**Fig. 1.**
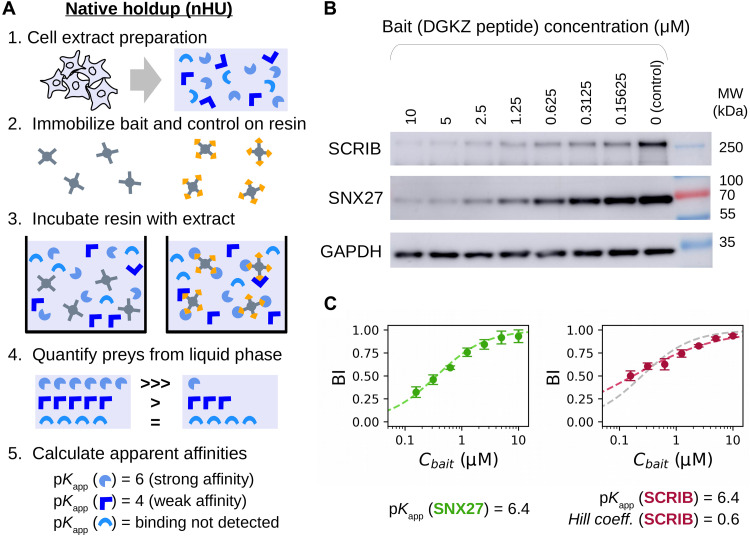
Principle and simple demonstration of nHU. (**A**) Schematic pipeline of nHU. Biotinylated baits and controls are immobilized on streptavidin resin at high concentration and are mixed with cellular extracts. After the binding equilibrium is reached, the liquid phase is separated by filtration or by centrifugation, and amounts of prey proteins are determined using standard protein analytical tools, such as WB or MS. The measured concentration ratio, in combination with the estimated amount of the immobilized bait concentration can be directly converted into apparent equilibrium dissociation constants (p*K*_app_). Note that the discarded resin from step 3 can be optionally processed as a regular pull-down experiment. (**B**) Demonstration of nHU titration experiment using the biotinylated PBM peptide of DGKZ as bait and biotin as control. Increasing amounts of bait-saturated resins were incubated with total Jurkat extracts. Supernatant fractions were probed with specific antibodies against endogenous full-length PDZ domain–containing proteins SCRIB and SNX27. (**C**) Results of nHU-WB experiments presented in (B). BI values of SNX27 (green, left) and SCRIB (red, right) were first fitted with a hyperbolic binding equation (dashed line). In the case of SCRIB, a significantly better fit was obtained using the Hill equation (red dashed line) compared to a hyperbolic binding equation (gray dashed line). Determined parameters are indicated below the plots. BI values were determined on the basis of three replicates. See fig. S1 and table S1 for additional data.

In nHU, total cell extracts are used as an analyte, and BI values of preys are measured with quantitative protein analytical methods, such as WB or MS (Fig. 1A) (*19*, *20*)⁠. While the qualitative proof of concept of nHU was demonstrated before, here, we develop it into a quantitative assay (*19*)⁠. Cell extracts contain thousands of distinct preys, all present at very low concentrations. We assume that the immobilized bait is in large excess relative to its potential preys in the extract so that even the cumulative amount of bound prey fractions should occupy only a negligible fraction of the immobilized bait. Together, the apparent equilibrium constants for interactions following a simple bimolecular binding mechanism can be calculated using the hyperbolic binding equation

$$K_{\mathrm{app}}=\frac{C_{\mathrm{bait}}-C_{\mathrm{bait}}*\mathrm{BI}}{\mathrm{BI}}$$ (2)

Through the study, we will report apparent affinities as negative logarithmic dissociation constant values (hence, p*K*_app_ = 6 corresponds to *K*_app_ = 1 μM, p*K*_app_ = 5 corresponds to *K*_app_ = 10 μM, etc.). The accuracy of the calculated affinities depends on the accuracy of the estimation of the concentration of the immobilized bait (*C*_bait_). This concentration needs to be higher than the cumulative concentration of all prey molecules of the extract that are prone to be captured by the bait. Previously, we determined the binding capacity of streptavidin resin (Streptavidin Sepharose High Performance, Cytiva) for various ligands by substituting affinities measured with orthogonal methods in binding equations, such as Eq. 2. As a rule of thumb, if 50 μl of bait-saturated streptavidin resin is mixed with 200 μl of extract, the estimated *C*_bait_ is between 5 and 20 μM, most likely around 10 μM (*12*, *21*)⁠.

### Measuring binding affinity with nHU coupled to WB

To investigate whether nHU can be used to measure affinities of a full-length protein directly from a cell extract, we addressed an already described interaction between the PDZ-binding motif (PBM) peptide of diacylglycerol kinase zeta (DGKZ) and full-length SNX27 endogenously present in Jurkat extract (*22*)⁠. First, we saturated streptavidin resin with biotin or biotinylated peptide. Then, we incubated total Jurkat cell extracts with various mixes of control and bait-saturated resin by keeping the resin/analyte ratio constant. This way, the concentration of SNX27 is fixed (determined by the lysate), whereas the concentration of the immobilized DGKZ peptide covers a wide range of concentration (*23*, *24*)⁠. The supernatant fractions of each experiment were assayed by WB using a specific antibody against SNX27 (Fig. 1B and fig. S1A). As expected, the measured BI values of SNX27 decreased when *C*_bait_ was decreased, following the hyperbolic binding equation (Eq. 2), revealing an apparent affinity of 6.4 p*K*_app_ (Fig. 1C, left, and table S1). This interaction between the PBM peptide of DGKZ and the isolated PDZ domain of SNX27 was already studied by both calorimetry and fragmentomic holdup, and the affinities were found to be 5.7 and 6.0 p*K*_d_, respectively (*12*, *22*)⁠. Thus, we found nHU to be a robust and versatile method for estimating biophysical properties of an endogenous full-length protein directly from cellular extract.

### Single-point nHU-WB to measure motif-protein affinities in medium throughput

**Fig. 2.**
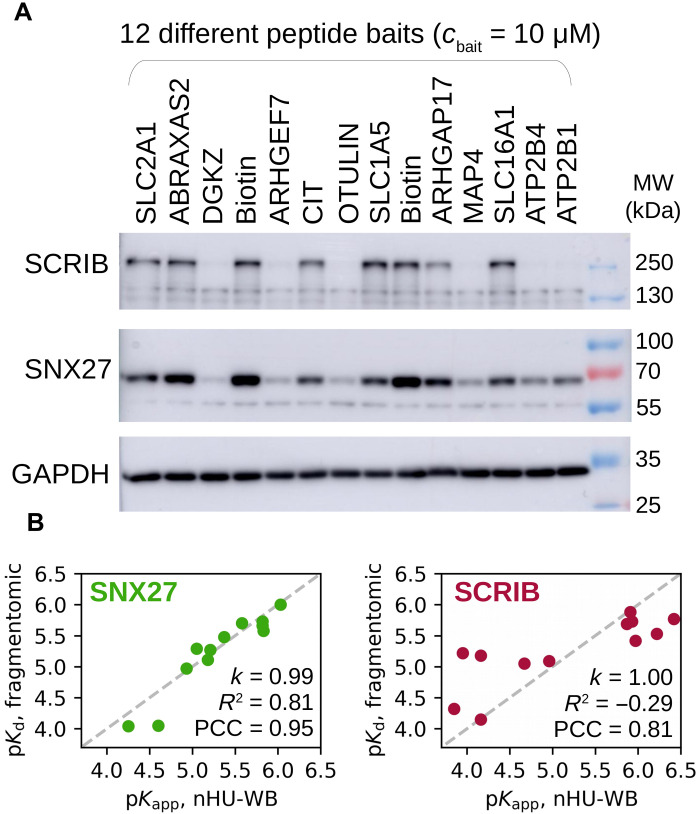
Single-point nHU for rapid apparent affinity measurements. (**A**) Demonstration of single-point nHU-WB using 12 different biotinylated PBM peptides (baits) or biotin (control) saturated streptavidin resin and total Jurkat extracts. Supernatant fractions were probed with specific antibodies against endogenous full-length PDZ domain–containing proteins SCRIB and SNX27. (**B**) Results of the nHU-WB experiment presented in (A). Correlation between in vitro fragmentomic affinities measured using PBM peptides and isolated PDZ domains (*12*)⁠ and apparent affinities measured with nHU between PBM peptides and full-length proteins. In the case of SCRIB (right), the site-specific fragmentomic affinities were combined assuming simple additivity. Direct proportionality was assumed between affinities (gray dashed line), and the coefficient of proportionality (*k*), coefficient of determination (*R*^2^) values, and Pearson correlation coefficient (PCC) values are indicated. Note the negative *R*^2^ value in the case of SCRIB, which indicates that a better fit could be obtained with a model with nonzero intercept; however, the physical basis of such model would be difficult to justify. Affinities were determined on the basis of three replicates. See fig. S1 and table S1 for additional data.

Titration experiments are key for investigating precise binding mechanism. However, they come with low throughput and high experimental cost. For this reason, we used single-point holdup experiments to measure apparent affinities of thousands of fragmentomic interactions at HTP in our previous study (*12*)⁠. Similarly, we propose that nHU experiments can also be performed using a single-bait concentration to probe binding equilibrium by assuming the binding mechanism. We used 12 different biotinylated 10-mer PBM peptides as baits or biotin as a negative control and performed nHU at a single-bait concentration (10 μM) using total Jurkat extracts as analyte (Fig. 2A and fig. S1B). We measured the depletion of SNX27 from the supernatants using nHU coupled to WB (nHU-WB), and the binding intensities of the PBM baits were converted into affinities using Eq. 2. The resulting affinities between full-length SNX27 and the 12 peptide baits showed excellent agreement with their fragmentomic affinities measured with the isolated PDZ domain of SNX27 with a Pearson correlation coefficient (PCC) of 0.95 (Fig. 2B, left, and table S1) (*12*)⁠.

However, not all interactions follow a simple binding mechanism (*25*)⁠. For example, Scribble (SCRIB) contains four PDZ domains that can synergize for the binding to PBMs, leading to interactions that are neither bimolecular nor single-sited. In addition to SNX27, we also measured the depletion of endogenous full-length SCRIB in the previous nHU-WB experiments. In the titration experiment, the affinity between the DGKZ peptide and full-length endogenous SCRIB was found to be more than an order of magnitude stronger than any of the site-specific affinities of its isolated domains (6.4 p*K*_app_ versus 5.1, 4.9, 4.7, and 4.4 p*K*_d_) (Fig. 1C, right, and table S1) (*26*)⁠. We also found that the interaction displays negative cooperativity with a Hill coefficient of 0.6 (*27*)⁠. Consequently, neither single-point holdup experiments nor Eq. 2 could reveal site-specific affinities of SCRIB with absolute confidence. Still, they could be used to calculate apparent affinities for ranking different interaction partners. Site-specific fragmentomic affinities can be combined to approximate an additive affinity of all interaction sites (*12*)⁠. Despite these rough approximations, a good correlation (PCC = 0.81) was found between the apparent affinities measured by single-point nHU and the combined fragmental affinities (Fig. 2B, right, and table S1). In addition, these single-point nHU experiments indicated that avidity between functional sites is markedly stronger when more isolated sites show detectable binding. In the case of motifs that show detectable binding with less than three isolated PDZ domains of SCRIB, calculated additive affinities were found to be systematically stronger than apparent affinities measured by nHU, possibly due to the negative cooperativity between the domains of SCRIB (fig. S2). In contrast, in motifs that show detectable binding with more than two isolated PDZ domains of SCRIB, calculated additive affinities were found to be weaker than the apparent affinities measured by nHU, indicating a large avidity between the binding sites. Still, single-point nHU experiments were found to be robust to explore intrinsic properties of a large number of interactions regardless of their binding mechanisms.

### nHU coupled to MS to measure domain-protein affinities proteome-wide

**Fig. 3.**
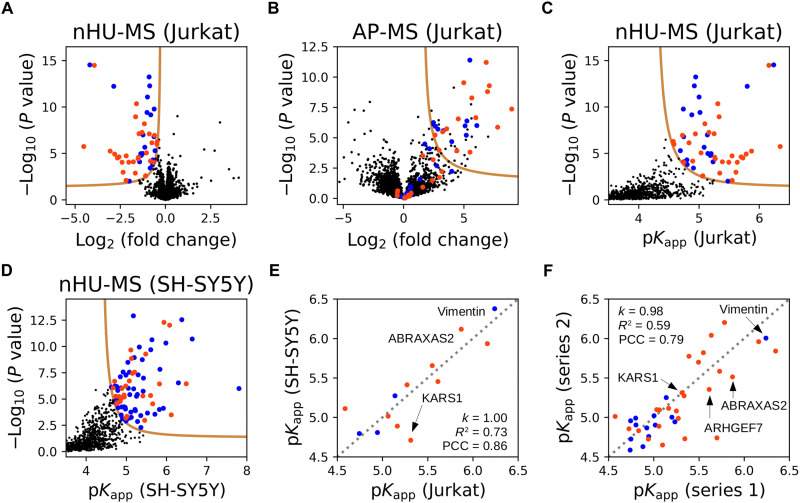
nHU-MS to survey affinities of the SNX27 PDZ domain proteome-wide. (**A**) Volcano plot of the nHU-MS experiment performed with SNX27_PDZ bait on total Jurkat extracts (*n* = 6). Identified interaction partners with or without C-terminal putative PBMs are colored with orange or blue, respectively. (**B**) Volcano plot of the control pull-down experiment performed on the leftover resin of the nHU experiment (*n* = 6). Preys are colored according to their coloring on (A). (**C** and **D**) Converted affinities of nHU experiments measured with Jurkat (C) or SH-SY5Y (D) extracts. (A to D) *P* values were calculated using a two-sided unpaired *t* test for nHU-MS and AP-MS experiments. The statistical significance thresholds for binding (tan lines) were determined at 1 σ and *P* value < 0.05. (**E**) Correlation between apparent affinities of SNX27_PDZ measured with Jurkat or SH-SY5Y extracts. (**F**) Correlation between apparent affinities of SNX27_PDZ measured in two independent experiment series using Jurkat extracts. The second experiment series was measured on a different MS instrument using different number of replicates (*n* = 3). Direct proportionality was assumed between affinities, and the coefficient of proportionality (*k*), *R*^2^ values, and PCC values are indicated in (E) and (F). Gray dashed line indicates the diagonal in (E) and (F). See fig. S3 and table S1 for additional data.

We coupled nHU to label-free quantitative MS to measure affinities of the SNX27 PDZ domain (SNX27_PDZ) for all the proteins quantifiable within a given cellular extract (Fig. 3A and table S1). The isolated PDZ domain fused to a biotinylated-His_6_-MBP tag was used as the bait, and the biotinylated-His_6_-MBP tag alone served as a control. To show the enrichment of preys that were depleted from the supernatant on the solid phase, the leftover resin after nHU was further processed according to a standard affinity-purification (AP) pull-down protocol (Fig. 3B).

We performed the nHU experiment using Jurkat cell extracts and assayed affinities of 3182 full-length endogenous proteins, of which 51 showed statistically significant depletion, most of which were also enriched on the resins in the pull-down assay (Fig. 3, A to C). In cases where there was a discrepancy between nHU coupled to MS (nHU-MS) and AP-MS, many false-positive preys of AP-MS showed no significant depletion in nHU, and similarly, many false-negative preys of AP-MS showed no significant enrichment during pull-down. We also repeated the experiment series using SH-SY5Y cell extracts and probed the affinities of 2076 full-length endogenous proteins, of which 83 showed statistically significant depletion (Fig. 3D and fig S3A). The determined apparent affinities for the 13 interaction partners of SNX27_PDZ that we quantified from both cell extracts were directly proportional with a PCC of 0.86 (Fig. 3E).

To further establish the repeatability and robustness of the nHU-MS assay, we repeated the experiments using a different mass spectrometer and six different PDZ domain baits taken from SCRIB (PDZ_1 and PDZ_2), discs large MAGUK scaffold protein 1 (DLG1, PDZ_1, and PDZ_2), Tax1 binding protein 3 (TAX1BP3), and SNX27 (fig. S3B and table S1). Using total Jurkat extract, we assayed 5595 endogenous proteins against these baits and identified 141 proteins that showed detectable interaction to at least one PDZ domain, and we quantified 198 affinities in total. Affinities measured using the SNX27_PDZ showed good agreement with the previous measurements done using extracts from the same cell type (PCC = 0.79, based on 39 common interaction partners) (Fig. 3F and table S1). Of the studied baits, the PDZ domain of SNX27 turned out to be the most promiscuous with 103 statistically significant interaction partners (while 180 SNX27 binding partners were identified in total on the basis of the three independent nHU-MS experiments). The other PDZ domains had lower promiscuity, and even the second most promiscuous PDZ domain (TAX1BP3) showed detectable binding with only 33 partners.

On the basis of all nHU-MS experiments, 216 proteins showed detectable interaction with at least one PDZ domain, and among these proteins, we identified 102 proteins with putative C-terminal PBMs. In our previous fragmentomic screen, the binding affinities of >400 isolated PBM peptides were already assayed against human PDZ domains (*12*)⁠. From the 102 full-length interaction partners found here, the PBMs of 39 had been addressed in our previous fragmentomic screen. Albeit this assay had quantified affinities between isolated PDZ domains and 10-mer C-terminal PBM peptides, they still showed weak correlation with the apparent affinities now measured by single-point nHU-MS with a PCC of 0.37 (fig. S4A and table S1). While some interactions of these isolated PDZ domains displayed very similar affinities when measured as a full-length protein from cellular extract with nHU or as a 10-mer C-terminal fragment, most interactions were found to be stronger in nHU experiments. On average, PDZ domains displayed >3-fold stronger apparent dissociation constants (∆p*K*_d_ ≈ 0.5) when measured against full-length proteins in nHU, as compared to their affinities for isolated PBM fragments.

### nHU-MS to measure protein-protein affinities proteome-wide

**Fig. 4.**
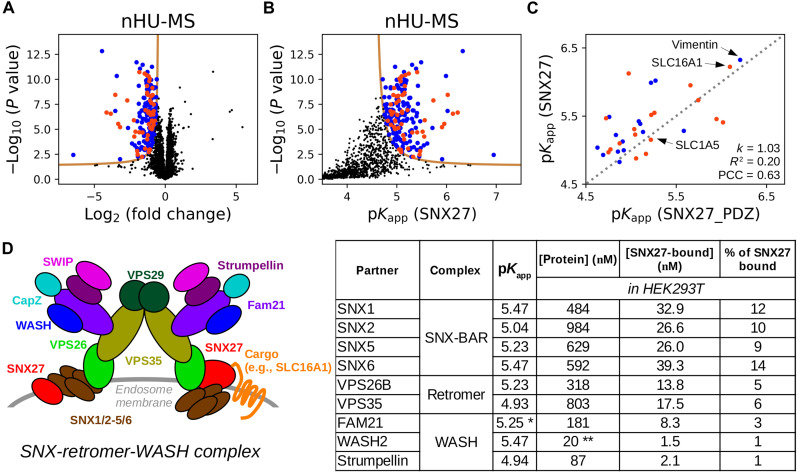
nHU-MS to survey affinities of full-length SNX27 proteome-wide. (**A** and **B**) nHU-MS experiment performed with recombinant SNX27 bait and total Jurkat extracts (*n* = 6) analyzed as a function of fold change (A) or converted affinities (B). Identified interaction partners with or without C-terminal putative PBMs are colored with orange or blue, respectively. *P* values were calculated using a two-sided unpaired *t* test, and statistical thresholds for binding (tan lines) were determined at 1 σ and *P* value < 0.05. (**C**) Correlation between apparent affinities of the SNX27_PDZ or full-length SNX27. Affinities of SNX27_PDZ were averaged on the basis of the three independent nHU experiment series. Direct proportionality was assumed between affinities, and obtained parameters as well as the diagonal (gray dashed line) are indicated. (**D**) Coarse topology, measured affinities, and estimated steady state of the SNX-retromer-WASH complex. The two hypothetical topological positions of SNX27 in the complex based on current observations were shown on the opposite sides of the dimeric complex. Measured affinities of SNX27 were combined with estimated total protein concentrations of HEK293T cells [taken from OpenCell (*35*)⁠] using the quadratic binding equation to estimate the amount of SNX27-bound complexes. Note that amounts of complexes of the same subcomplexes are in the same regime: ~10 to 15% of the total amount of SNX27 found in HEK293T cells (280 nM) is bound to SNX-BARs, ~5% of SNX27 is bound to retromer, and ~2% of SNX27 is bound to WASH. *Affinity determined for FAM21 showed statistical significance below threshold. **The concentration of WASH2 in HEK293T is unknown and is substituted with the concentration of WASH1. See fig. S3 and table S1 for additional data.

We performed an nHU-MS experiment using total Jurkat extracts as analyte and recombinant full-length SNX27 fused to an N-terminal biotinylated-MBP and C-terminal His_6_ tag as a bait or biotinylated-His_6_-MBP tag alone as a control (Fig. 4, A and B, and table S1). In addition to the PDZ domain, full-length SNX27 contains a Phox homology and a FERM (4.1, ezrin, radixin, moesin) domain. We quantified 3128 full-length endogenous preys, of which 198 showed statistically significant depletion in nHU. Most of these targets were also enriched in a subsequent control pull-down experiment (fig. S3C). Among the interaction partners of SNX27, 32 proteins were also identified in the previous nHU experiments as partners of SNX27_PDZ, including 17 with putative C-terminal PBMs, and the obtained affinities correlate with a PCC of 0.63 (Fig. 4C).

Affinities obtained from nHU can originate from either direct or indirect interactions through large complexes (fig. S4B). We identified the retromer complex as an interaction partner of SNX27, with the strongest affinity measured with VPS26B (*28*, *29*)⁠. We also detected the association of SNX27 and the heterodimeric SNX1/2-SNX5/6 SNX-BAR complex with the strongest affinity measured with SNX1. A short fragment of SNX1/2 was reported to interact with the FERM domain of SNX27 with comparable affinity to the one obtained from nHU (*30*)⁠. We identified the actin-regulatory Wiskott-Aldrich syndrome protein and scar homolog (WASH) complex as the partner of SNX27 with the strongest affinities measured with WASH2 and FAM21 (*31*)⁠. The 1.5-mDa multi-tRNA synthetase complex cofractionates with the retromer complex in extracts, and we found that all components have detectable affinity with SNX27_PDZ with the strongest interaction with KARS1 (*32*)⁠. We identified the 320-kDa BRCC36 isopeptidase (BRISC) complex as a target of SNX27_PDZ with the strongest affinity measured with ABRAXAS2 (*33*)⁠. Last, we also detected the oligomeric small guanosine triphosphatase regulator GIT-PIX complex as the interaction partner of SNX27_PDZ with ARHGEF7 (also called β-PIX) displaying the strongest affinity (*34*)⁠. In the case of the last three complexes, which were identified as the partner of SNX27_PDZ, only the partners with the highest affinities had PBMs satisfying the SNX27_PDZ consensus. These examples may suggest that when large complexes are captured by a bait in nHU assays, the subunit that binds the bait directly displays the strongest measured affinity, while weaker apparent affinities of other subunits can be an asset to map topologies of the complexes.

Apparent affinities obtained from nHU experiments can be used to estimate steady states of networks in various conditions by combining with cellular concentrations of proteins. We took apparent affinities between SNX27 and other components of the SNX-retromer-WASH complex and combined them with estimated total protein concentrations measured for human embryonic kidney (HEK) 293T cells using the quadratic binding equation (Fig. 4D) (*35*)⁠. On the basis of this coarse analysis, 10% of cellular SNX27 is expected to bind to SNX-BARs, 5% to the retromer complex, and 2.5% to the WASH complex in HEK293T cells.

### Interactions of SNX27 with membrane and filamentous proteins

**Fig. 5.**
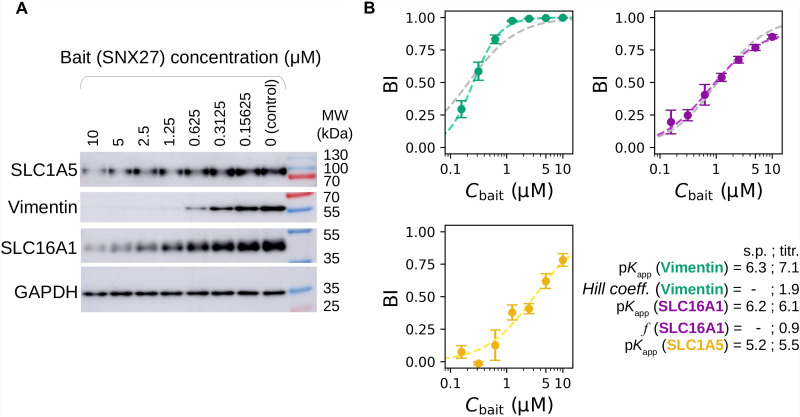
Exploring binding mechanisms of SNX27 interactions with nHU. (**A**) Results of nHU-WB titration experiments performed with full-length SNX27 bait. (**B**) Endogenous full-length SLC1A5 (yellow), SLC16A1 (purple), and Vimentin (green) prey depletions were quantified using specific antibodies. BI values of all prey were first fitted with a hyperbolic binding equation. In the case of SLC1A5, good fit was achieved with a simple hyperbolic binding equation (yellow dashed line). In the case of SLC16A1, an imperfect fit was achieved with a simple hyperbolic binding equation (gray dashed line), and more accurate fit was found when 10% inactive fraction was assumed (purple dashed line, *f* = 0.9, see Materials and Methods for further details). In the case of Vimentin, a low-quality fit was achieved with a simple hyperbolic binding equation (gray dashed line), and near-perfect fit was obtained using the Hill equation (green dashed line). Equilibrium affinities of single-point measurements (s.p.) and parameters determined from the titration experiments (titr.) are indicated in the bottom-right corner. BI values were determined on the basis of three replicates. See fig. S6 and table S1 for additional data.

For further mechanistic characterization, we selected three representative full-length interaction partners of SNX27 that displayed similar affinities with full-length SNX27 or SNX27_PDZ baits (table S1 and fig. S5). The monocarboxylate transporter 1 (SLC16A1) (*36*)⁠ and the intermediate filament-forming Vimentin (*37*)⁠ were among the strongest partners of SNX27, and the neutral amino acid transporter B(0) (SLC1A5) (*38*)⁠ displayed weaker affinity. In the nHU-MS experiment performed with the six isolated PDZ domains, SLC16A1 and SLC1A5 showed detectable interaction with only SNX27_PDZ, and Vimentin showed detectable interaction with four different PDZ domains, yet SNX27_PDZ was its strongest binder (table S1). To explore the binding mechanisms of their interactions, we performed an nHU-WB titration experiment with full-length SNX27 (Fig. 5, fig. S6, and table S1). We have found that SLC1A5 followed a simple binding mechanism (as in Eq. 2). Although SLC16A1 also followed a similar binding mechanism, approximately 10% of the detected protein seemed to be incapable of binding possibly because of regulatory modifications of its C-terminal tail segment. In the case of filamentous Vimentin, we observed a positive cooperative binding mechanism resulting in a stronger SNX27 binding affinity than that approximated from single-point measurements (*27*)⁠. However, despite the more complex binding mechanisms of Vimentin and SLC16A1, their apparent affinities estimated from single-point nHU experiments were still indicative of their strong interaction with SNX27. It is worth mentioning that characterizing interactions of full-length transmembrane or filamentous proteins with traditional quantitative biochemical methods is highly challenging yet seems to be easily addressed with nHU.

**Fig. 6.**
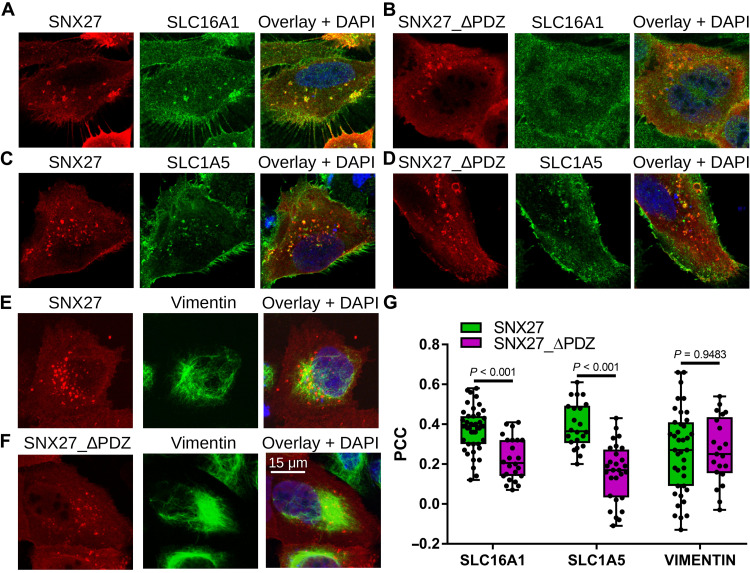
Colocalization of SNX27 and SNX27_∆PDZ with selected partner proteins identified with nHU. (**A** to **F**) Representative colocalization images of U2OS cells expressing hemagglutinin (HA)–tagged SNX27 (A, C, and E) or HA-tagged SNX27_ΔPDZ (B, D, and F) stained with anti-HA antibody (red) and antibodies against endogenous SLC16A1 (A and B), SLC1A5 (C and D), or Vimentin (E and F) (green), and with DAPI (blue). Confocal images are shown for the two transmembrane SLC transporters, and a maximum intensity projection is shown for Vimentin. (**G**) Colocalization was quantified on confocal images for each transfected cell (*n* > 20) by measuring intensity correlation (PCC). Box plots indicate the median and upper and lower quartiles, and whiskers label the minimal and maximal measured PCC values. Individual data points representing measurements of single cells are also indicated. *P* values were calculated using two-sided unpaired *t* tests. See fig. S7 for additional confocal images.

To investigate whether the observed intrinsic binding properties of the above-investigated interactions lead to the formation of their complex in cells, we measured colocalization (Fig. 6 and fig. S7). We immunostained endogenous interaction partners of SNX27 in U2OS cells expressing hemagglutinin (HA)–tagged full-length or truncated SNX27 lacking its PDZ domain (SNX27_ΔPDZ). Both SNX27 constructs were found to be enriched in endomembrane structures. These SNX27 foci were mostly found in the proximity of Vimentin filaments; however, this was found to be independent of the SNX27 PDZ domain possibly because of the endogenous SNX27 background or because of other interactions (Fig. 6, E and F). While Vimentin was not enriched in these SNX27 foci, both SLC16A1 and SLC1A5 were (Fig. 6, A to D). Moreover, both SLC transporters showed statistically significantly stronger colocalization with SNX27 than with SNX27_ΔPDZ. Therefore, these results indicate that while these two transmembrane SLC transporter proteins are cargos of the SNX27-retromer complex, Vimentin is not and may instead contribute to other activities of the SNX-retromer complex that will require further inspection.

### Limitations of the nHU assay

Apparent affinities, estimated by either single-point or titration nHU experiments, contain uncertainty from multiple sources. First of all, the relative concentration measurements, which are essential to determine BI values, are affected by the precision of the selected analytical method. For example, the robustness of determined BI values is affected by the sensitivity and specificity of antibodies in nHU-WB experiments or by the abundances and other characteristics of the prey proteins in nHU-MS. While these parameters greatly affect the precision of the assay, the accuracy of converted affinities mostly depend on the bait concentration. Since at the moment, there is no way to measure this parameter experimentally, and we only estimate its value, affinities quantified by nHU should also be considered as estimates. However, affinities measured from the same nHU experiment have a constant bait concentration, and therefore, BI ranking of preys should strictly follow their affinity ranking. Despite this, we have found that we can get a practical estimation of the error of the estimated affinities if we compare results of multiple datasets, e.g., by comparing PCC values. For a more detailed discussion about sources of errors and their propagation to the estimated affinities, see Materials and Methods and fig. S8.

On the basis of nHU titration experiments, we obtained binding models other than hyperbolic in several occasions. Positive or negative cooperative mechanism assumes that a single bait molecule can interact with multiple prey molecules, where the binding affinities of subsequent binding events are increased or decreased, respectively. Since nHU experiments are carried out at large bait excess, it is rather unusual to observe such mechanisms, yet the presence of negative or positive cooperativity may indicate multivalent interactions (like in the case of SCRIB) or oligomeric prey molecules (like Vimentin), respectively. In addition to cooperative binding, partial activity was also observed. When single-point nHU experiments are carried out and we are lacking any a priori information about such mechanisms, Ockham’s razor tells us to assume the simplest binding model for affinity conversion. However, this way, we introduce error in the conversion, affecting the resulting affinities in various directions. If a partial activity is ignored, we will always underestimate the real affinities. In contrast, if cooperative mechanisms are ignored, we can both over- and underestimate the real affinities depending on both the type of mechanism (negative or positive cooperativity) and the relative ratio of the bait concentration and the prey affinity.

The dynamic range of the nHU assay is also related to the errors of concentration measurements, as well as to the bait concentration. Experimental determination of BI values is most accurate when the prey depletion is large enough to be robustly measured but small enough that the amount of the leftover prey is still measurable. In other words, if BI values are close to the extremes (close to 0 or 1), the affinity conversion is less reliable. Therefore, the dynamic range of BI measurements highly depends on the sensitivity and the robustness of the selected analytical method. Since the affinity conversion of these BI values only depends on the bait concentration (in case of a simple mechanism), the dynamic range of nHU in affinity units of measurement can only be estimated. As a guide, users of nHU need to pay attention to the quality of prey quantification and modify the bait concentration accordingly. By changing the bait concentration, for example, by changing the resin/analyte ratio or by mixing bait-saturated resin with control resin at various ratios, the BI values can be increased or decreased. Last, to measure very high affinities, one needs to decrease the amount of the bait and to dilute the analyte for maintaining the excess of the bait. Such condition reduces the accuracy of the protein concentration measurement and therefore the affinity estimation, and at a certain point, the nHU assay will only be capable of providing an estimation of the upper limit of affinities.

## DISCUSSION

Although common interactomic assays are efficient for interaction screening, most of them do not measure biophysical properties of interactions. Pull-down–based approaches were used in several ways to gain quantitative insight into affinities of interactions. For example, measured “stoichiometric” ratios of baits and preys have been used in immunoprecipitation experiments as a proxy to discriminate between “strong” and “weak” complexes (*39*)⁠. Recently, we have also shown that in parallel pull-down experiments using various baits, the relative enrichment values of endogenous preys correlate with their corresponding fragmentomic affinities (*12*)⁠. Pull-down methods were even used to directly estimate affinities of baits using measured enrichment values, yet the consequences of washing steps were not considered (*40*, *41*)⁠. Other types of methods have also been developed to measure affinities directly from cell extracts, but these are mostly low throughput and require high expertise (*42*–*46*)⁠. Therefore, we still lack a robust method to measure affinities from cell extracts, and most HTP affinity measurements are limited to labor-intensive fragmentomic approaches that require expensive reagents and instruments.

Here, we introduced nHU as a versatile tool to measure apparent equilibrium affinities proteome-wide of recombinant or synthetic baits. Although these affinities can be indirect and can be perturbed by protein heterogeneity, nHU experiments can give us insight into an interactomic dimension that was never reached at this scale before: affinities of full-length proteins and even large complexes directly obtained from cell extracts. The resulting affinities are coherent between experiments and even between cell extracts and may provide better reproducibility for large-scale interactomic studies in the future. Compared to qualitative pull-down–based methods, nHU involves less experimental steps and robustly ranks identified targets by their observed affinities. It can be equally used to cost-efficiently screen affinities across the proteome by using single-point measurements and to accurately investigate binding mechanisms using titration experiments. All mature protein analytical technologies can be used in combination with nHU, such as antibody-based approaches like routine WB or label-free MS. In principle, nHU is not limited to studying the interactions of proteins, and both baits and preys can be molecules of different kinds. Overall, nHU experiments can be effortlessly implemented in most laboratories and could greatly advance the exploration of the quantitative human affinity interactome.

## MATERIALS AND METHODS

### Peptide and recombinant protein preparation

Biotinylated peptides were chemically synthesized on an ABI 443A synthesizer with a standard Fmoc strategy with the biotin group attached to the N-terminus via a trioxatridecan-succinamic acid (ttds)linker and were purified with high-performance liquid chromatography (LC) (>95% purity). All C-terminal PBM peptides were 10 amino acid long (10-mer). Predicted peptide masses were confirmed by MS. Peptide concentrations were determined on the basis of their dry weight.

SNX27_PDZ (40-141) was cloned as His_6_-AviTag-MBP-TEV-SNX27_PDZ, and full-length SNX27 (1-541) was cloned as AviTag-MBP-SNX27-His_6_ construct in pET vectors. The empty His_6_-AviTag-MBP-TEV vector was used to produce biotinylated MBP for control experiments. Proteins were coexpressed with BirA biotin ligase (PET21a-BirA, Addgene, no. 20857) in *Escherichia coli* BL21(DE3) cells. At isopropyl-β-d-thiogalactopyranoside (IPTG) induction (1 mM IPTG at 18°C overnight), 50 μM biotin was added to the media. Harvested cells were lysed in a buffer containing 50 mM tris (pH 7.5), 150 to 300 mM NaCl, 50 μM biotin, 2 mM β-mercaptoethanol, cOmplete EDTA-free protease inhibitor cocktail (Roche, Basel, Switzerland), 1% Triton X-100, and trace amount of deoxyribonuclease, ribonuclease, and lysozyme. Lysates were frozen at −20°C before further purification steps. Lysates were sonicated and centrifuged for clarification. Expressed proteins were captured on in-house prepacked Ni-IDA (Protino Ni-IDA Resin, Macherey-Nagel, Duren, Germany) columns and were washed with at least 10 column volumes of cold wash buffer [50 mM tris (pH 7.5), 150 mM NaCl, and 2 mM β-mercaptoethanol] before elution with 250 mM imidazole. The Ni elution was collected directly on a preequilibrated amylose column (amylose high flow resin, New England Biolabs, Ipswich, MA). Amylose column was washed with 5 column volumes of cold wash buffer before fractionated elution in a buffer containing 25 mM Hepes (pH 7.5), 150 mM NaCl, 1 mM TCEP, 10% glycerol, 5 mM maltose, and cOmplete EDTA-free protease inhibitor cocktail. The concentration of proteins was determined by their ultraviolet absorption at 280 nm before aliquots were snap-frozen in liquid nitrogen and stored at −80°C.

### Cell cultures and extract preparation for the nHU experiment

SH-SY5Y cells [American Type Culture Collection (ATCC), no. CRL-2266, RRID: CVCL_0019] were grown in RPMI 1640 (Gibco) medium completed with 10% fetal calf serum (FCS) and gentamicin (40 μg/ml), diluted 1:5 every 3rd/4th day. U2OS cells (ATCC, no. HTB-96, RRID: CVCL_0042) were grown in Dulbecco’s modified Eagle’s medium [Gibco, glucose (1 g/liter)] completed with 10% FCS and gentamicin (40 μg/ml), and diluted 1:10 every 3rd/4th day. Jurkat E6.1 cells [European Collection of Authenticated Cell Cultures (ECACC), no. 88042803, RRID: CVCL_0367] were grown in RPMI 1640 (Gibco) medium completed with 10% FCS and gentamicin (40 μg/ml), and diluted 1:12 every 3rd/4th day. All cells were kept at 37°C and 5% CO_2_.

To prepare seminative total cell extracts, cells were seeded on T-175 flasks. After they reached confluency, adherent cells were washed with phosphate-buffered saline (PBS) once and collected by scraping with ice-cold lysis buffer [50 mM Hepes-KOH (pH 7.5), 150 mM NaCl, 1% Triton X-100, 1× cOmplete EDTA-free protease inhibitor cocktail, 2 mM EDTA, 5 mM TCEP, and 10% glycerol]. Jurkat cells were collected by 1000*g* × 5 min centrifugation, washed once with PBS, and then collected by 1000*g* × 5 min centrifugation again and lysed in ice-cold lysis buffer. Lysates were sonicated 4 × 20 s with 1-s-long pulses on ice and then incubated rotating at 4°C for 30 min. Lysates were centrifuged at 12,000 rpm 4°C for 20 min, and the supernatant was kept for further analysis. Total protein concentration was measured by the standard Bradford method (Bio-Rad protein assay dye reagent, no. 5000006) using a bovine serum albumin (BSA) calibration curve (MP Biomedicals, no. 160069, diluted in lysis buffer) on a Bio-Rad SmartSpec 3000 spectrophotometer instrument. Lysates were diluted to 2 mg/ml concentration and were snap-frozen in liquid nitrogen and stored at −80°C until measurement. Note that different lysate-preparing protocols can lead to different pools of binding-capable proteins, and therefore, in some cases, it may be essential to modify the above-described protocol, e.g., by removing EDTA from the lysis buffer to measure interactions mediated by metal ions.

### Resin preparation and nHU experiment

For saturating streptavidin resin with biotinylated peptides or biotin, 50 μl of streptavidin resin was mixed with biotin or peptide at 40 to 60 μM concentration in 6 to 6.5 resin volume for 60 min. To saturate streptavidin resin with biotinylated proteins, 50 μl of streptavidin resin (Streptavidin Sepharose High Performance, Cytiva) was mixed with biotinylated MBP or MBP-PDZ at 40 to 50 μM concentration in 20× resin volume for 60 min. After saturation, resins were washed a single time [10 resin volume, holdup buffer: 50 mM tris (pH 7.5), 300 mM NaCl, and 1 mM TCEP, 0.22-μm filtered] and were depleted with biotin (10 resin volume, 10 min, holdup buffer supplemented with 100 μM biotin). Last, resins were washed two times (10 resin volume, holdup buffer).

For single-point nHU experiments carried out at ~10 μM bait concentration, 50 μl of saturated streptavidin resin was mixed with 200 μl of cell lysate (2 mg/ml). Titration experiments were carried out by mixing control and bait-saturated resin and keeping the total resin volume constant; e.g., ~5 μM bait concentration can be achieved by mixing 25 μl of bait-saturated streptavidin resin with 25 μl of control resin and 200 μl of cell lysate (2 mg/ml). Control and bait-saturated resins were prepared in larger amounts, and a serial dilution was prepared with these presaturated resins to achieve different resin ratios.

Unless specified elswhere, the nHU mixture was incubated at 4°C for 2 hours. After the incubation ended, the resin was separated from the supernatant by a brief centrifugation (15 s, 2000 g). Then, half of the supernatant was removed by pipetting without any delay to avoid any resin contamination; e.g., 100 μl of supernatant is collected if 200 μl of cell lysate was used as analyte. Alternatively, the supernatant can be centrifuged an additional time to clarify it further, removing any possible resin contamination. In principle, the separation of the supernatant should be as fast as possible since any delay can perturb the equilibrium. In practice, we did not observe changes in measured BI values if we recovered the supernatant within the first minute after centrifugation, possibly because the perturbation of equilibrium is, in part, a diffusion-limited process. Alternatively, the resin can be separated from the supernatant using filter plates (e.g., various products of Millipore, Burlington, MA) or spin columns (e.g., Pierce spin cups—cellulose acetate filter from Thermo Fisher Scientific, Waltham, MA) to achieve faster separation.

Since exceptionally strong complexes may have extremely slow dissociation rate constants that make it highly difficult to reach binding equilibrium (*24*)⁠, we also verified that some interactions indeed reached binding equilibrium using our standard protocol (2 hours of incubation) by probing nHU-WB experiments using various incubation times (fig. S5B). We used SNX27_PDZ bait and performed nHU with Jurkat lysates using incubation times of 15, 30, 120, and 240 min. Then, nHU supernatants were probed with WB for SLC1A5, SLC16A1, and Vimentin. For these interaction partners, we did not observe significant change by prolonging the nHU reaction in the monitored time frame compared to our standard protocol. Note that the nHU assay was originally referred to as “pure-crude holdup assay from eukaryotic cells” when its first qualitative proof of concept was demonstrated (*19*)⁠.

### Affinity purification

Leftover beads from nHU-MS experiments were washed three times immediately after the separation of the supernatant [10 resin volume in buffer containing 50 mM tris (pH 8.5), 150 mM NaCl, 1% Triton X-100, 10× cOmplete EDTA-free protease inhibitor cocktail, 2 mM EDTA, and 1 mM TCEP]. Then, the beads were washed two times [10 resin volume buffer containing 50 mM tris (pH 8.5), 150 mM NaCl, and 1 mM TCEP]. Last, captured protein was eluted from the resin in two steps, and the eluted fractions were pooled. For each elution, the beads were incubated for 30 min with three resin volume buffer containing 20 mM tris (pH 8.5), 100 mM NaCl, 500 μM TCEP, and 8 M urea. Between each step, the beads were separated by mild centrifugation, and the supernatant was removed by gentle pipetting.

### Western blot

nHU samples were mixed with 4× Laemmli buffer [120 mM tris-HCl (pH 7), 8% SDS, 100 mM dithiothreitol, 32% glycerol, 0.004% bromphenol blue, and 1% β-mercaptoethanol] in a 3:1 ratio. Equal amounts of samples were loaded on 8 or 10% acrylamide gels. Transfer was done into PVDF membranes using a Trans-Blot Turbo Transfer System and a Trans-Blot Turbo RTA Transfer kit (Bio-Rad, no. 1704273). After 1 hour of blocking in 5% milk, membranes were incubated overnight at 4°C in primary antibody in 5% milk. The following antibodies and dilutions were used: anti-SCRIB (1:1000, Thermo Fisher Scientific, no. PA5-54821, RRID: AB_2647030), anti-SNX27 (1:1000, Thermo Fisher Scientific, no. MA5-27854, RRID: AB_2735367), anti–glyceraldehyde-3-phosphate dehydrogenase (GAPDH, 1:5000, Sigma-Aldrich azide-free version of AB_2924240), anti-SLC16A1 (1:1000, Sigma-Aldrich, no. HPA003324, RRID: AB_1856982), and anti-Vimentin (1:1000, CST, no. 5741, clone D21H3, RRID: AB_10695459). Membranes were washed three times with tris-buffered saline (TBS)–Tween and incubated at room temperature for 1 hour in secondary antibody [Jackson ImmunoResearch, peroxidase-conjugated Affinipure goat anti-mouse (H + L), no. 115-035-146 RRID: AB_2307392 and goat anti-rabbit (H + L), no. 111-035-003 RRID: AB_2313567] in 5% milk (concentration 1:10,000). After washing three times with TBS-Tween, membranes were exposed to chemiluminescent horseradish peroxidase substrate (Immobilon, no. WBKLS0100) and revealed in a docking system (Amersham Imager 600, GE). Densitometry was carried out on raw Tif images by using Fiji ImageJ 1.53c. Between different primary antibody labeling, the membranes were either exposed to 15% H_2_O_2_ to remove secondary signal (in the case of different species) or stripped with mild stripping buffer [glycine (15 g/liter), SDS (1 g/liter), and 1% Tween 20 (pH 2.2)] to remove primary signal (in the case of same species).

### Sample digestion for MS

The nHU samples were precipitated with trichloroacetic acid (TCA) 20% overnight at 4°C and centrifuged at 14,000 rpm for 10 min at 4°C. The protein pellets were washed twice with 1 ml of cold acetone and air-dried. The protein extracts were solubilized in 8 M urea, reduced with 5 mM TCEP for 30 min, and alkylated with 10 mM iodoacetamide for 30 min in the dark. Double digestion was performed at 37°C with 500 ng of endoproteinase Lys-C (Wako, Richmond, USA) for 4 hours, followed by fourfold dilution and an overnight digestion with 500 ng of trypsin (Promega, Charbonnieres les Bains, France). Peptide mixtures were then desalted on a C18 spin-column and dried on a speed vacuum.

### LC tandem MS analysis

Samples were analyzed using an Ultimate 3000 nano-RSLC coupled in line via a nano-electrospray ionization source, with an LTQ-Orbitrap ELITE mass spectrometer (Thermo Fisher Scientific, San Jose, CA) or with the Orbitrap Exploris 480 mass spectrometer (Thermo Fisher Scientific, Bremen, Germany) equipped with a high field asymmetric ion mobility spectrometry (FAIMS) module. Peptide mixtures were injected in 0.1% trifluoroacetic acid on a C18 Acclaim PepMap100 trap column [75 μm inner diameter (ID) × 2 cm, 3 μm, 100 Å, Thermo Fisher Scientific] for 3 min at 5 μl/min with 2% acetonitrile (ACN) and 0.1% formic acid (FA) in H_2_O and then separated on a C18 Acclaim PepMap100 nano-column (75 μm ID × 50 cm, 2.6 μm, 150 Å, Thermo Fisher Scientific) at 300 nl/min and 40°C with a 90-min linear gradient from 5 to 30% buffer B (A: 0.1% FA in H_2_O/B: 80% ACN, 0.1% FA in H_2_O) and regeneration at 5% B. Spray voltage was set to 2.1 kV and heated capillary temperature was set to 280°C.

For the Orbitrap Elite, the mass spectrometer was operated in positive ionization mode in data-dependent mode with survey scans from mass/charge ratio (*m*/*z*) 350 to 1500 acquired in the Orbitrap at a resolution of 120,000 at *m*/*z* 400. The 20 most intense peaks from survey scans were selected for further fragmentation in the linear ion trap with an isolation window of 2.0 Da and were fragmented by collision-induced dissociation (CID) with a normalized collision energy of 35% (TOP 20 CID method). Unassigned and single-charged states were excluded from fragmentation. The ion target value for the survey scans (in the Orbitrap) and the MS2 mode (in the linear ion trap) were set to 1E6 and 5E3, respectively, and the maximum injection time was set to 100 ms for both scan modes. Dynamic exclusion was set to 20 s after one repeat count with mass width at ±10 parts per million (ppm).

For the Orbitrap Exploris 480 MS associated with the FAIMS module, a combination of two compensation voltages, −40 and −55 V, was chosen with a cycle time of 1 s for each. For the full MS1 in data-dependent acquisition (DDA) mode, the resolution was set to 60,000 at *m*/*z* 200 and with a mass range set to 300 to 1400. The full MS AGC target was 300% with an IT set to auto mode. For the fragment spectra in MS2, AGC target value was 100% (standard) with a resolution of 30,000 and the maximum injection time set to auto mode. Intensity threshold was set at 1E4. Isolation width was set at 2 *m*/*z*, and normalized collision energy was set at 30%. All spectra were acquired in centroid mode using positive polarity. Default settings were used for FAIMS with voltages applied as described previously, and with a total carrier gas flow set to 4.2 liters/min.

### MS data analysis

Proteins were identified by database search using SequestHT (Thermo Fisher Scientific) with Proteome Discoverer 2.4 software (PD2.4, Thermo Fisher Scientific) on the human FASTA database downloaded from UniProt (reviewed, release 2021_06_03, 20380 entries, https://uniprot.org/, complemented with sequences of likely contaminants, such as MBP, streptavidin, or trypsin). Precursor and fragment mass tolerances were set at 7 ppm and 0.6 Da, respectively, and up to two missed cleavages were allowed. For the data acquired on the Orbitrap Exploris 480, the software Proteome Discoverer 2.5 version was used with a human FASTA database from UniProt (reviewed, release 2022_02_21, 20291 entries). Precursor and fragment mass tolerances were set at 10 ppm and 0.02 Da, respectively, and up to two missed cleavages were allowed. For all the data, oxidation (M, +15.995 Da) was set as variable modification and carbamidomethylation (C, + 57.021 Da) was set as fixed modification. Peptides and proteins were filtered with a false discovery rate at 1%. Label-free quantification was based on the extracted ion chromatography intensity of the peptides. All samples were measured in technical triplicates. The measured extracted ion chromatogram (XIC) intensities were normalized on the basis of median intensities of the entire dataset to correct minor loading differences. For statistical tests and enrichment calculations, nondetectable intensity values were treated with an imputation method, where the missing values were replaced by random values similar to the 10% of the lowest intensity values present in the entire dataset. Unpaired two-tailed *t* test, assuming equal variance, was performed on obtained log_2_ XIC intensities. All raw LC tandem MS data have been deposited to the ProteomeXchange via the PRIDE database with identifiers PXD034790 and PXD036024.

### Statistics

Since the goal of nHU experiments is to measure affinities instead of identifying interaction partners of high confidence, technical repeats were preferred to measure the occurring changes in protein concentrations more precisely over measuring imprecise affinities from multiple independent nHU experiments. In the case of single-point nHU-MS experiments, one or two independent experiments were performed with three technical replicates (total *n* = 3 or 6), and mean intensities were used for fold change calculations. In the case of nHU-WB experiments, WBs were repeated three times to minimize the error of densitometric quantification, and mean values of determined BI values were used for affinity calculations. Since the same cell extract of known concentration was used for nHU-WB experiments, all samples were handled identically, and equal volumes of extracts were loaded on the acrylamide gels; GAPDH was only used to verify WB loading qualitatively in nHU-WB experiments. Similar affinities were obtained when densitometric GAPDH levels were used for normalization; however, the results showed higher SDs likely because of the inclusion of an additional source of variability.

Measured BI values of nHU-WB experiments were fitted using the hyperbolic binding equation

$$\mathrm{BI}=\frac{C_{\mathrm{bait}}}{\left(K_{\mathrm{app}}+C_{\mathrm{bait}}\right)}$$ (3)

where *C*_bait_ is the immobilized bait concentration and *K*_app_ is the apparent dissociation constant. (Note that Eq. 2 is just a rearranged version of Eq. 3.) In the case of the nHU-WB experiment between full-length SNX27 and SLC16A1, a BI offset was observed with the hyperbolic fit, and a partial activity was assumed using the function

$$\mathrm{BI}=f\frac{C_{\mathrm{bait}}}{\left(K_{\mathrm{app}}+C_{\mathrm{bait}}\right)}$$ (4)

Here, the *f* factor was found to be 0.9, indicating that only 90% of the quantified SLC16A1 protein pool shows binding activity. In cases where the hyperbolic equation could not reach a reasonable solution because of cooperative effects, the Hill equation was used for fitting

$$\mathrm{BI}=\frac{C_{\mathrm{bait}}^{n_{H}}}{\left(K_{\mathrm{app}}+C_{\mathrm{bait}}^{n_{H}}\right)}$$ (5)

where *n*_H_ is the Hill coefficient (*n* > 1 for positive and *n* < 1 for negative cooperative interactions). Fitting was performed in QtiPlot using standard procedures (scaled Levenberg-Marquardt algorithm with 1000 iterations) and figures were generated with custom Python scripts using Matplotlib.

Affinities for each detected protein were calculated in nHU-MS experiments where the experimental BI was a positive value using Eq. 2; however, only those were considered for subsequent analysis where statistical robustness was observed. For each detected protein in nHU-MS experiments, a *P* value was calculated on the basis of the measured intensities of samples (*n* = 6) and controls (*n* = 6) using a two-tailed unpaired Student’s *t* test. For each nHU-MS and AP-MS experiment series, a hyperbolic binding threshold was calculated taking into account both measured intensities and the general distribution of the entire dataset, similarly as described in other works (*4*)⁠. This threshold was calculated for AP-MS as follows

$$y=y_{0}+\frac{c}{\left(x-x_{0}\right)}$$ (6)

and for nHU-MS experiments as

$$y=y_{0}+\frac{-c}{\left(x+x_{0}\right)}$$ (7)

where *y* is the *P* value threshold at fold change of *x*, *c* is a curvature parameter empirically fixed at 1 for nHU-MS and 4 for AP-MS experiments, *y*_0_ is the minimal *P* value threshold, and *x*_0_ is the minimal fold change value for any given dataset. The minimal *P* value was defined at 1.3 −log_10_(*P*), and thus, there is at least 95% probability that any identified interaction partners are true interaction partners. The minimal fold change value cutoff was set at 1 σ and was determined by measuring the width of the normal distribution of all measured fold changes in a given experiment. Note that this threshold can only be interpreted for interaction partners with fold change values of −*x > x*_0_ in the case of nHU-MS experiments or *x > x*_0_ in the case of AP-MS experiments.

For simplicity, no multiple testing correction was applied. However, other statistical thresholding can also be used to minimize false discoveries. For example, multiple testing procedures, such as the Benjamini-Hochberg method, can be used to define an adjusted significance threshold value corresponding to 1 or 5% false discovery rate. Then, either this threshold can be considered as a standalone *P* value threshold, or it can replace the *y*_0_ *P* value offset parameter in the hyperbolic binding threshold (Eq. 7). This way, type I error can be greatly minimized, yet type II error can increase.

Future users of nHU need to decide in accordance with their project to use or not to use such more stringent thresholds. They need to keep in mind that although they will obtain a more reliable list of binders with less false positives, at the same time, they may inevitably filter many interactions where the assay accurately quantified affinities. Measuring affinities of weak and transient interactions using modest bait concentration will result in low BI values, which, combined with the low precision of protein quantification, can yield low *P* values. However, neither a low *P* value nor a high one is indicative of the accuracy of the affinity measurement.

Although SDs of measured BI values can be calculated from replicates, it is challenging to propagate these errors to affinities. To do this in an analytical way, one has to first consider how the uncertainty of either or both total and free prey concentrations affect the uncertainty of BI values

$$\sigma \mathrm{BI}\approx \sqrt{{(\frac{\partial \mathrm{BI}}{\partial C_{\mathrm{free}}}*\sigma C_{\mathrm{free}})}^{2}+{(\frac{\partial \mathrm{BI}}{\partial C_{\mathrm{total}}}*\sigma C_{\mathrm{total}})}^{2}}$$ (8)

which can be simplified if concentrations are normalized (*C*_total, normalized_ = 1)

$$\sigma \mathrm{BI}\approx \sigma C_{\mathrm{free},\mathrm{normalized}}+C_{\mathrm{free},\mathrm{normalized}}*\sigma C_{\mathrm{total},\mathrm{normalized}}$$ (9)

Note that the uncertainty of the total amount of prey concentration mostly affects the overall uncertainty when the free concentration is close to the total (i.e., when the BI value is small). Error in the measurement of the BI values and errors of the estimated bait concentrations also propagate to the error of affinities

$$\sigma K_{\mathrm{app}}\approx \sqrt{{(\frac{\partial K_{\mathrm{app}}}{\partial C_{\mathrm{bait}}}*\sigma C_{\mathrm{bait}})}^{2}+{(\frac{\partial K_{\mathrm{app}}}{\partial \mathrm{BI}}*\sigma \mathrm{BI})}^{2}}=\sqrt{{\left(\frac{(1-\mathrm{BI})*\sigma C_{\mathrm{bait}}}{\mathrm{BI}}\right)}^{2}+{\left(\frac{-C_{\mathrm{bait}}*\sigma \mathrm{BI}}{{\mathrm{BI}}^{2}}\right)}^{2}}$$ (10)

where the assumed binding model was taken from Eq. 2. In principle, for each binding mechanism, the partial derivatives need to be determined. Unfortunately, at the moment, there is no direct way to measure neither the bait concentration nor its SD. On the basis of previous investigations, we have found that by following our protocols, the bait concentration can change over a twofold range in either direction. Bait concentration may also depend on the size of the immobilized protein in cases where a bound bait molecule could block or limit the binding of additional biotinylated bait molecules to neighboring streptavidin-binding sites by causing steric hindrance; however, this effect may only become substantial if the size of the bait reaches a critical value. Note that in case one compares affinities from a single experiment, the value of the bait concentration is constant through the experiment (with σ*C*_bait_ = 0), and therefore, incorrect estimation of this value only causes a shift in estimated affinities. As a result, even in the absence of accurate bait concentrations, the ranking of measured BI values of partners identified from a single nHU experiment will match their affinity ranking. To visualize this error propagation, we used simulated data with random error in one of these parameters, or in a combination of parameters on fig. S8. Last, in the case of nHU titration experiments, fitting error may also greatly contribute to the error of the estimated affinity, depending on inaccurate models and experimental noise of protein quantification.

While BI measurements define the overall precision of any holdup assay, the affinity accuracy mainly depends on the bait concentration. At the moment, this parameter is only estimated on the basis of previous observations (*12*)⁠. In future generations of the nHU, more direct methods are needed to measure the error of this estimation that can be used to calculate affinities at high accuracy. For these reasons, we do not specify propagated errors for the calculated affinities since they would be misleading because absolute affinities could change with a more accurate bait concentration. However, affinity ranking of different prey measured from the same nHU experiment will be unaffected by such transformation. For example, on the basis of our experiments, we can conclude that the true affinity of SNX27 with Vimentin is stronger than with SLC16A1, and the affinity with SLC16A1 is stronger than with SLC1A5, but it is possible that these affinities can diverge systematically compared to the reported affinities.

### Calculating amounts of complex formations

Determined affinities can be combined with total protein concentrations to estimate amounts of binary complexes. These coarse predictions can be performed for any cellular proteomes, even at subcellular resolutions. We performed such calculations to estimate amounts of SNX-retromer-WASH complexes bound to SNX27 using estimated protein concentrations previously measured for HEK293T cells (*35*)⁠. In principle, such absolute proteomic datasets could also be estimated from the control nHU experiments directly using straightforward tools such as the proteomic ruler; however, one has to assume that the prepared cellular extract is representative of the entire cellular proteome of the monitored cell. In these calculations, one cannot use the hyperbolic binding equations (Eq. 2 or Eq. 3) since the concentrations of binding partners are comparable, and the partial binding occupation of SNX27 cannot be neglected. Instead, we used the quadratic binding equation (or similar) to make predictions

$$[AB]=\frac{({\left[A\right]}_{\mathrm{tot}}+{\left[B\right]}_{\mathrm{tot}}+K_{d})-\sqrt{{\left({\left[A\right]}_{\mathrm{tot}}+{\left[B\right]}_{\mathrm{tot}}+K_{d}\right)}^{2}-4*({\left[A\right]}_{\mathrm{tot}}*{\left[B\right]}_{\mathrm{tot}})}}{2}$$ (11)

where [AB] is the concentration of the complex under equilibrium, [A]_tot_ and [B]_tot_ are the total concentrations of the binding partners (e.g., quantified with absolute proteomics), and *K*_d_ is the steady-state dissociation constant. Calculated amounts of complexes were also converted into percentage of SNX27 bound by comparing the amounts of complexes with the total SNX27 concentration (280 nM). Note that although such calculations can be performed for PDZ-mediated interactions, results will be flawed because of their mutually exclusive nature. Future rule-based network-level calculations should consider affinities and concentrations of all PDZ and PBM proteins, as well as their binding mechanisms, to estimate amounts of all possible complexes in the network.

### Immunostaining

For transient transfection, the full-length SNX27 (1 to 541) or SNX27_ΔPDZ (140 to 541) constructs were cloned in mammalian pCI vector containing N-terminal HA tag for immunolabeling. For detection of protein localization, 0.25 × 10^5^ U2OS cells per well were seeded onto a coverslip-containing 24-well plate. The next day, cells were transfected with HA-tagged constructs using JetPRIME reagent (Polyplus) and, 24 hours after transfection, were washed once and fixed for 15 min with 4% formaldehyde solution, permeabilized for 10 min with 0.3% Triton X-100 in PBS at room temperature, and blocked for 1 hour in 5% BSA and 0.3% Triton X-100 in PBS at room temperature. Staining of HA, SLC1A5, SLC16A1, and Vimentin was performed overnight at 4°C using anti-HA (1:750, BioLegend, no. 901502, RRID: AB_2565007), anti-SLC1A5 (1:200, Abcam, no. ab237704, clone CAL33, RRID: not BSA/azide free version of AB_2924240), anti-SLC16A1 (1:200, Sigma-Aldrich, no. HPA003324, RRID: AB_1856982), and anti-Vimentin (1:500, CST, no. 5741, clone D21H3, RRID: AB_10695459), respectively. After three washes with PBS, secondary antibodies were used for 1 hour at room temperature in 5% BSA and 0.3% Triton X-100 in PBS: Alexa Fluor 594–conjugated anti-mouse (1:1000; Invitrogen, no. A-11032, RRID: AB_2534091) and Alexa Fluor 488–conjugated anti-rabbit (1:1000; Invitrogen, no. A-11034, RRID: AB_2576217). Cover glasses were mounted to microscopy slides by Vectashield mounting medium with 4′,6-diamidino-2-phenylindole (DAPI) (Vector laboratories, Burlingame, CA). Images were taken using a Leica SP5 confocal microscope (Leica Camera AG, Wetzlar, Germany) with an HCX PL APO 63×/1.40 to 0.60 oil objective using excitation at 405 nm (diode), 488 nm (Argon laser), and 594 nm (HeNe laser) and emission at 415 to 480, 510 to 560, and 610 to 695 nm for DAPI, Alexa 488, and Alexa 594, respectively. Images were processed by the Fiji ImageJ software. In every image, transfected cells based on HA signal were selected as regions of interest manually, and Coloc 2 plugin was used to determine single-cell PCC values. Statistics and box plots were done using GraphPad Prism 7 software.

## Notes

View/request a protocol for this paper from *Bio-protocol*.
