# Supplementary material for: Native holdup (nHU) to measure binding affinities from cell extracts
Source: Sci Adv. 2022 Dec 21;8(51):eade3828. doi: 10.1126/sciadv.ade3828 (PMC9770967; doi:10.1126/sciadv.ade3828)
Supplement: Supplementary file 1 — Figs. S1 to S8 [file sciadv.ade3828_sm.pdf]

Science Advances
AAAS

Supplementary Materials for

Native holdup (nHU) to measure binding affinities from cell extracts

Boglarka Zambo *et al.*

Corresponding author: Gilles Trave, travegi@igbmc.fr; Gergo Gogl, goglg@igbmc.fr

Sci. Adv. **8**, eade3828 (2022)
DOI: 10.1126/sciadv.ade3828

The PDF file includes:

Figs. S1 to S8

Other Supplementary Materials for this manuscript includes the following:

Table S1

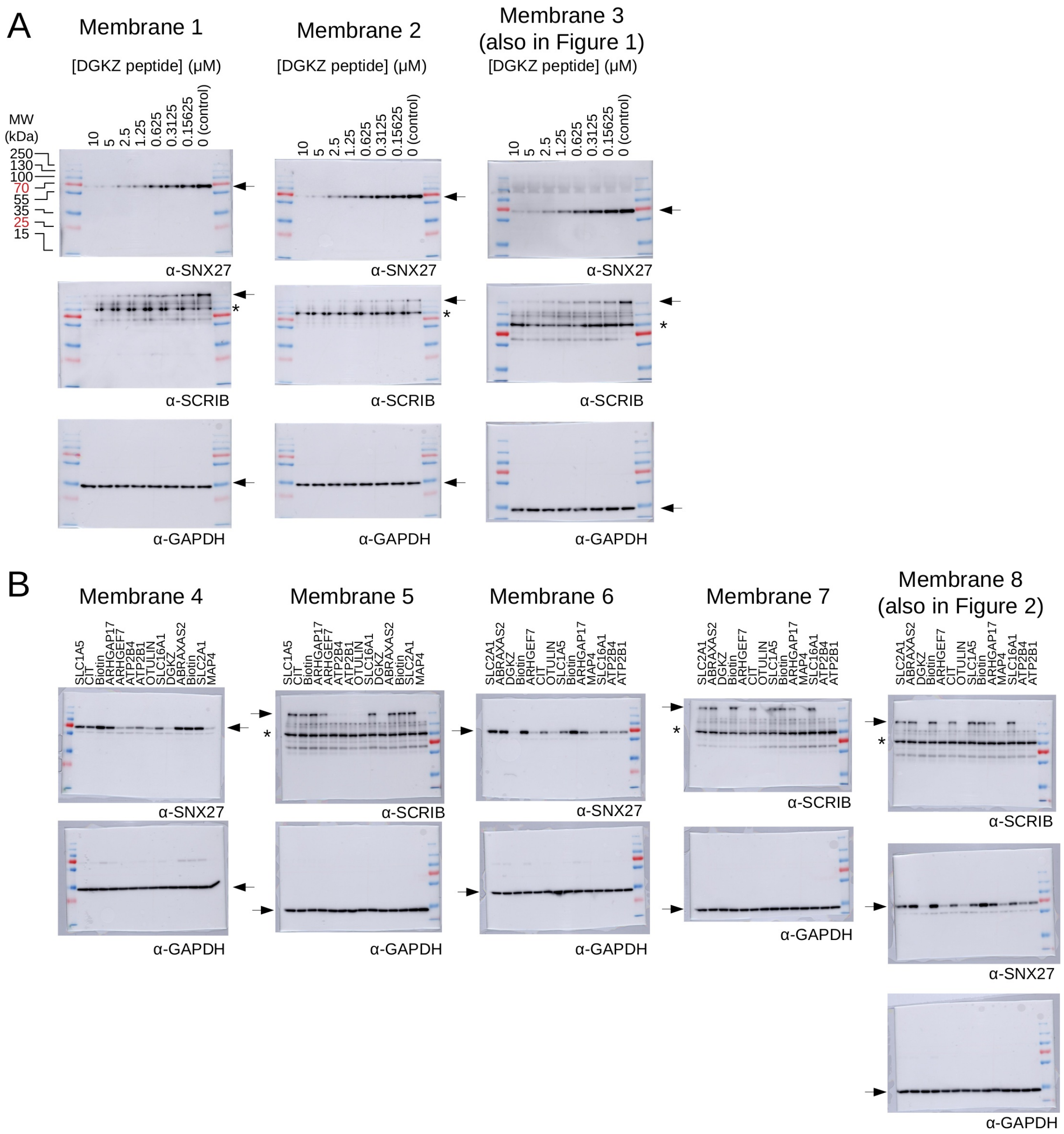

Supplementary Figure 1, Raw results of nHU-WB experiments using peptide baits. Overlay of luminescent signal (specific signal) with visible image (MW ladder) of all blots used for analysis of nHU-WB experiments using peptide baits. Membranes were H2O2 treated between SCRIB and SNX27 or SCRIB and GAPDH antibodies. Membranes were mildly stripped between SNX27 and GAPDH antibodies. Asterisk indicates a known aspecific band obtained on western blots labeled with SCRIB antibody. Primary antibodies and their working concentrations are indicated in the bottom right corner. (see Methods for further details)

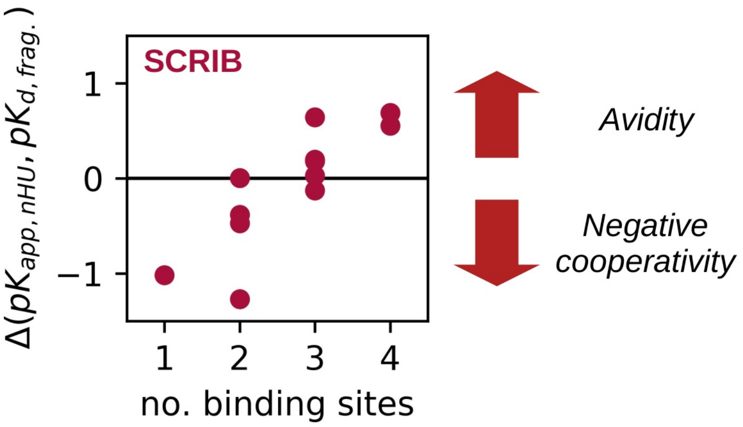

Supplementary Figure 2, The combination of nHU and fragmentomics can help to reveal how multiple interaction sites work together via avidity and cooperativity. Differences between calculated additive fragmentomic affinities and affinities measured by nHU plotted in the function of the number of PDZ domains that give measurable fragmentomic affinities out of the 4 isolated domains of SCRIB. Note that the the fragmentomic affinities of the least promiscuous SCRIB_PDZ4 domain was not measured with all studied peptide motifs. The data indicates that when a partner can detectably interact with one or two interaction sites of SCRIB, then negative cooperative effects dominate these interactions and the resulting affinity appears to be weaker in nHU. In contrast, when three or all four interaction sites show strong fragmentomic affinities, they form a very avid complex that counteracts the negative cooperativity, just as in the case of the DGKZ peptide. See Figure 1 and 2, Supplementary Figure 1 and Supplementary Table 1 for additional data.

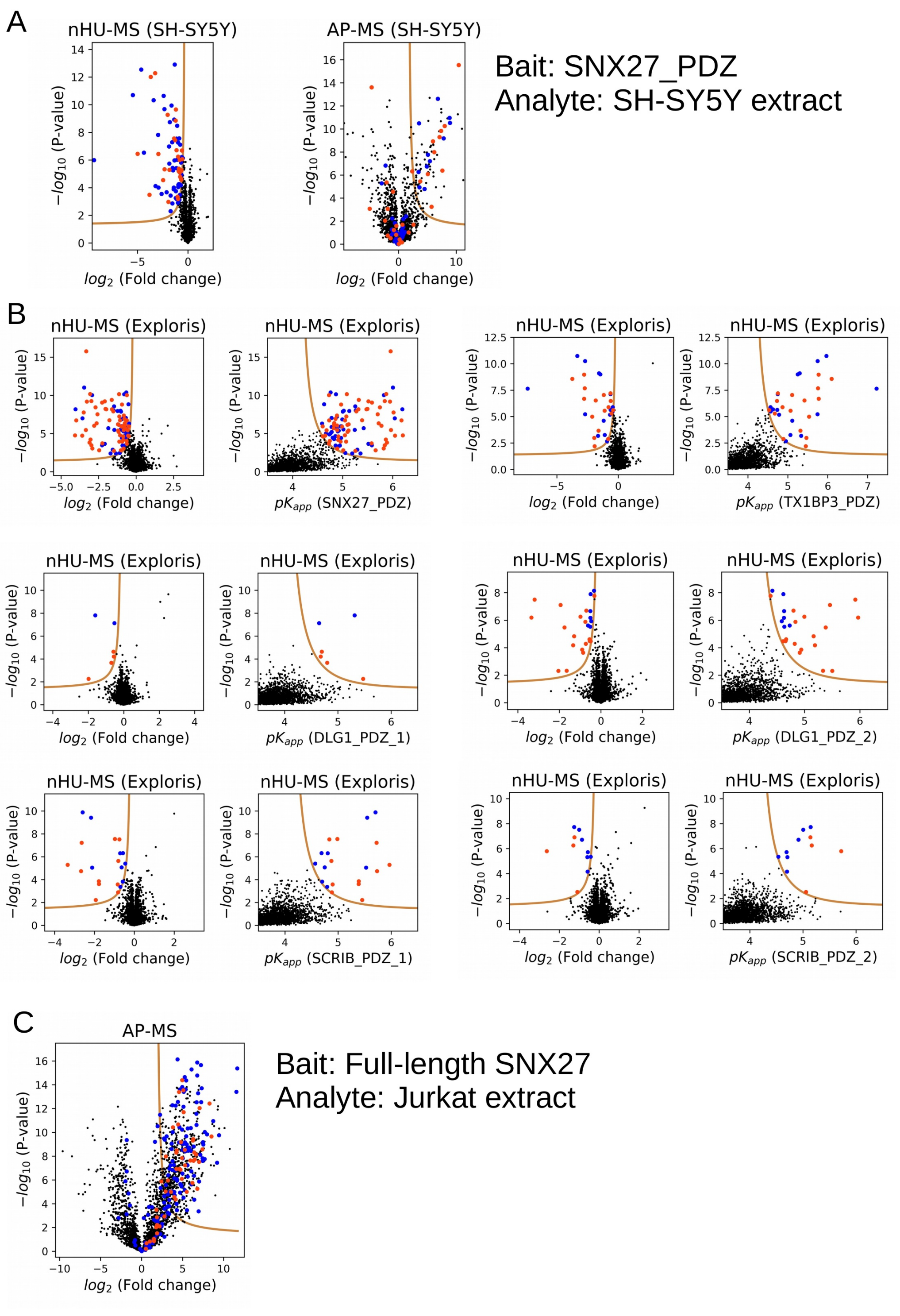

Supplementary Figure 3, Additional data about nHU-MS and AP-MS experiments. (A) Volcano plot of the nHU-MS (left) and AP-MS (right) experiments performed with recombinant SNX27_PDZ bait and total SH-SY5Y cell extracts (n=6). Converted affinities of the nHU-MS experiments are shown in Figure 3D. (B) Volcano plot of the nHU-MS (left) and converted affinities (right) of the nHU-MS experiment series carried out with 6 different PDZ domain baits (n=3). (C) Volcano plot of the control pulldown experiment performed on the leftover resin of the nHU experiment showed in Figure 4 (n=6). Identified interaction partners with and without putative C-terminal PBM motifs are colored in red and blure, respectively. The coloring of preys in AP-MS experiments is identical as on their corresponding nHU experiment (see Figure 4A for reference of panel C).

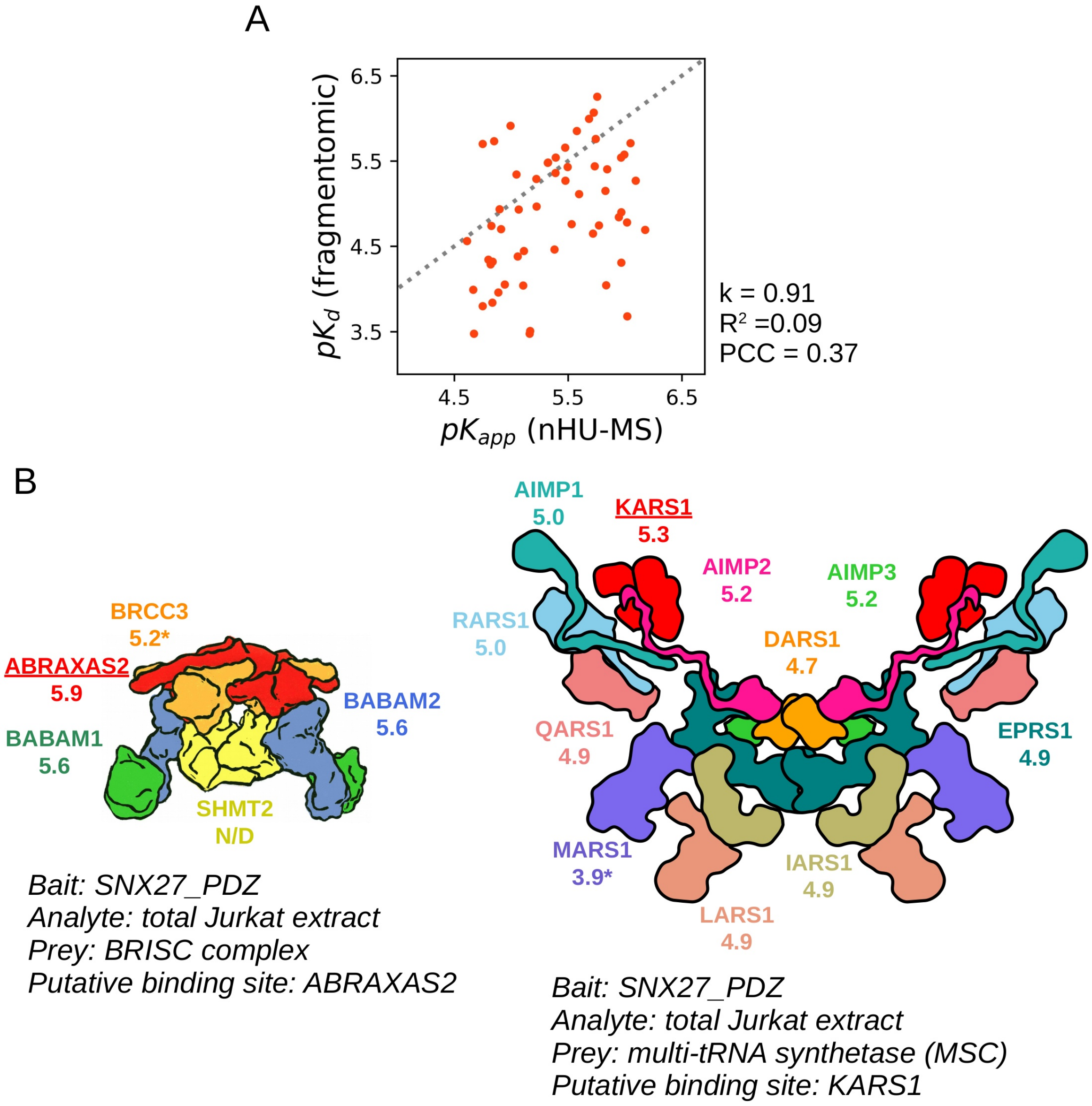

Supplementary Figure 4, Additional information for nHU-MS experiments. (A) Correlation between *in vitro* fragmentomic affinities measured using PBM peptides and the isolated PDZ domain (*12*) and apparent affinities measured with nHU between the same domains and full-length proteins. In case of SNX27, the average apparent affinities are used based on the three nHU-MS experiment series. (B) Affinities of two large multi-subunit complexes measured with nHU-MS. The BRISC deubiquitinase complex (left) was found to interact with SNX27 with the exception of the inhibitory subunit SHMT2. Almost all component of the multi-tRNA-synthetase complex (right) was found to interact with SNX27. The subunits with expected direct interactions are underlined on both complexes. For all subunits of the presented complexes, the indicated affinities were obtained with the SNX27_PDZ bait from Jurkat extracts (experiment sreies 1). Asterisk indicates that the affinity was quantified but was found to be not statistically robust. N/D indicates that while the protein amount was quantified, no affinity could be determined from the experiment. See Supplementary Table 1 for additional data.

A

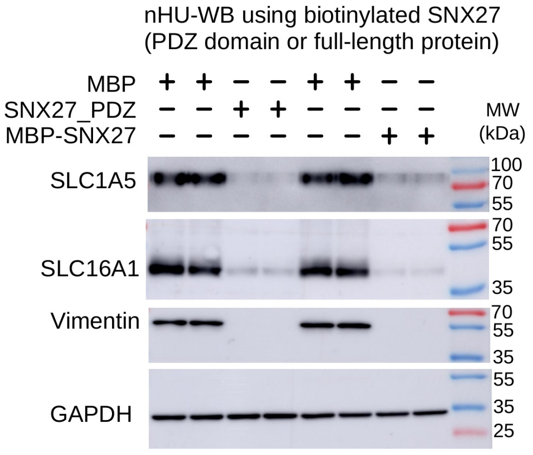

prey	bait	Measured pK_{app} (Jurkat)
SLC1A5	SNX27_PDZ	5.71
	SNX27	5.47
SLC16A1	SNX27_PDZ	5.78
	SNX27	6.07
Vimentin	SNX27_PDZ	>6
	SNX27	>6

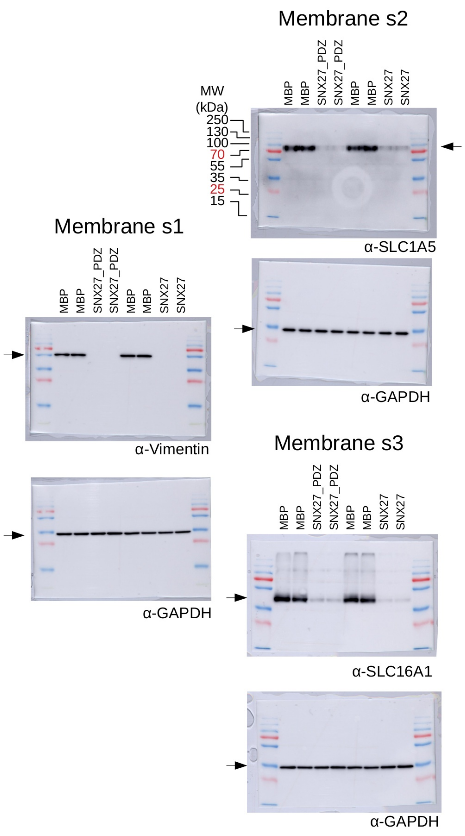

B

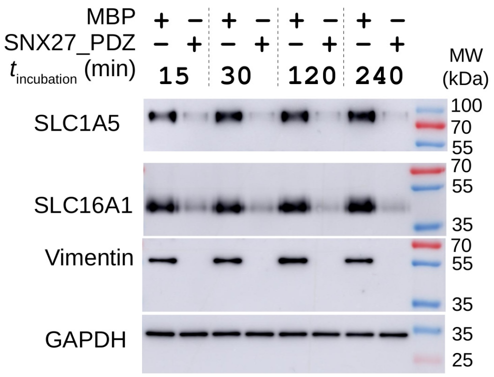

	Measured BI @ incubation time			
	15 min	30 min	120 min	240 min
**SLC1A5**	0.75	0.80	0.80	0.87
**SLC16A1**	0.65	0.69	0.80	0.81
**Vimentin**	>0.95	>0.95	>0.95	>0.95

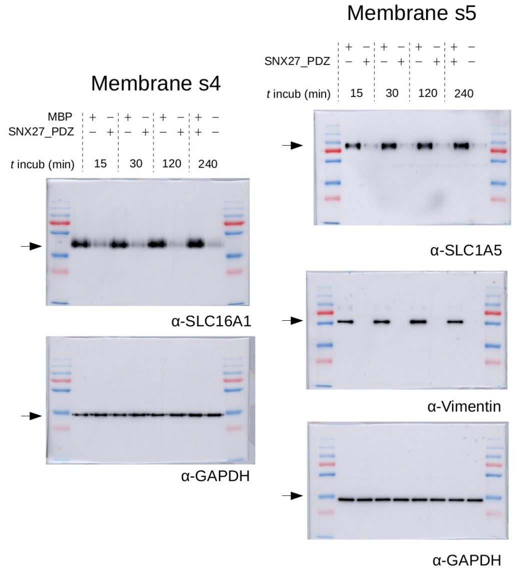

Supplementary Figure 5, Additional nHU-WB experiments.
(A) nHU-WB experiments using SNX27 and SNX27_PDZ baits and total Jurkat extracts. 2 independent experiments are analyzed on the same membrane and the reported affinity was calculated from their averages. (B) nHU-WB experiments performed using SNX27_PDZ bait and Jurkat cell extracts, measured at step-wise increasing incubation times. No detectable change was observed with the different incubation times in the case of Vimentin and only a small change was detected after 30 min incubation in the case of SLC16A1. In all other nHU experiments of our study, 120 min incubation times was applied. Bait concentration was 10 µM in both experiment series. On the right side of both panels, raw results of the western blot experiments are shown similarly to Supplementary Figure 1, or 5. Membranes were H2O2 treated before GAPDH blotting and mildly stripped between SLC1A5 and vimentin in case of membrane s5.

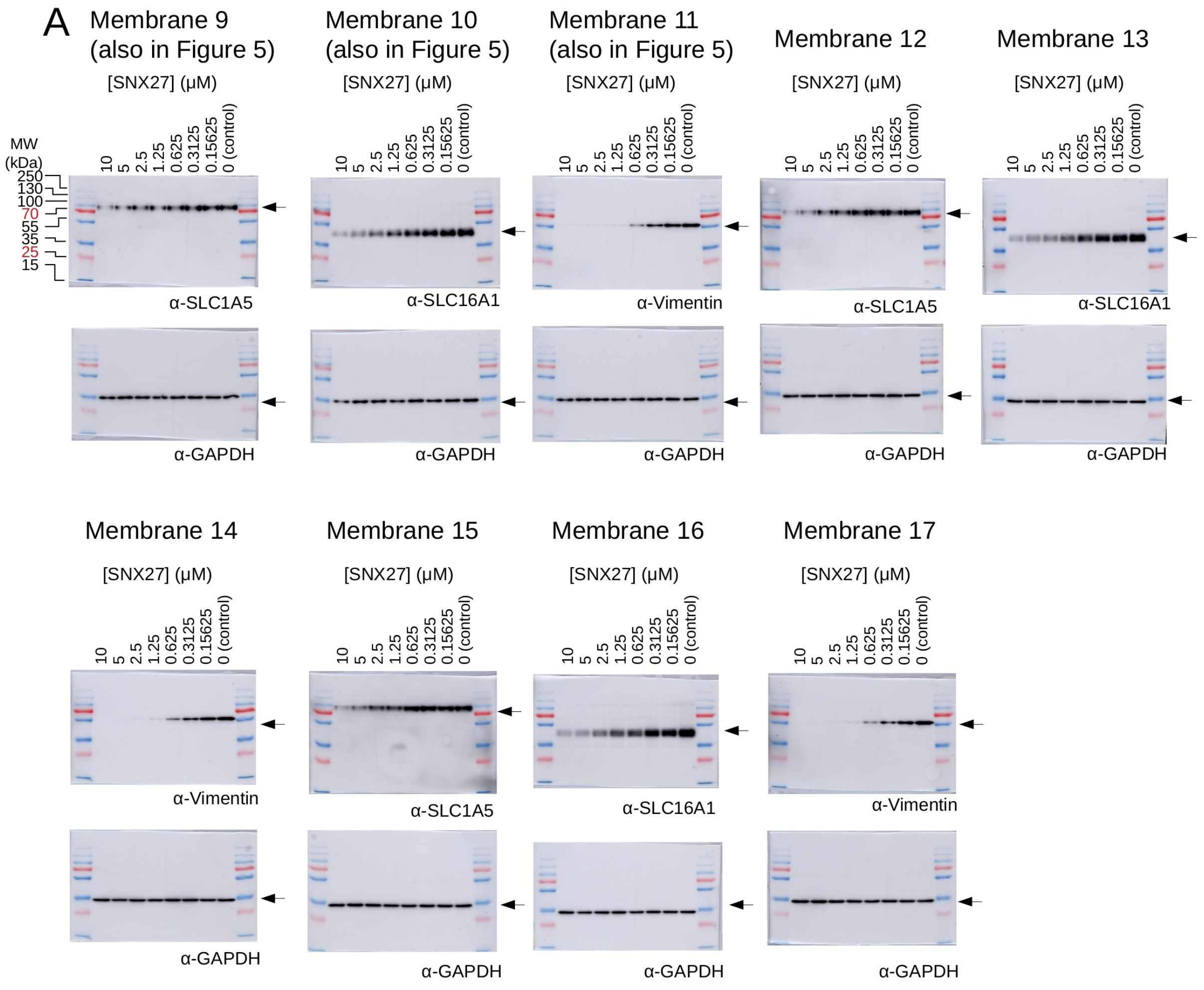

Supplementary Figure 6, Raw results of nHU-WB experiments using SNX27. Overlay of luminescent signal (specific signal) with visible image (MW ladder) of all blots used for analysis of nHU-WB experiments using SNX27 baits. Membranes were H_2O_2 treated before GAPDH blotting. Primary antibodies and their working concentrations are indicated in the bottom right corner.

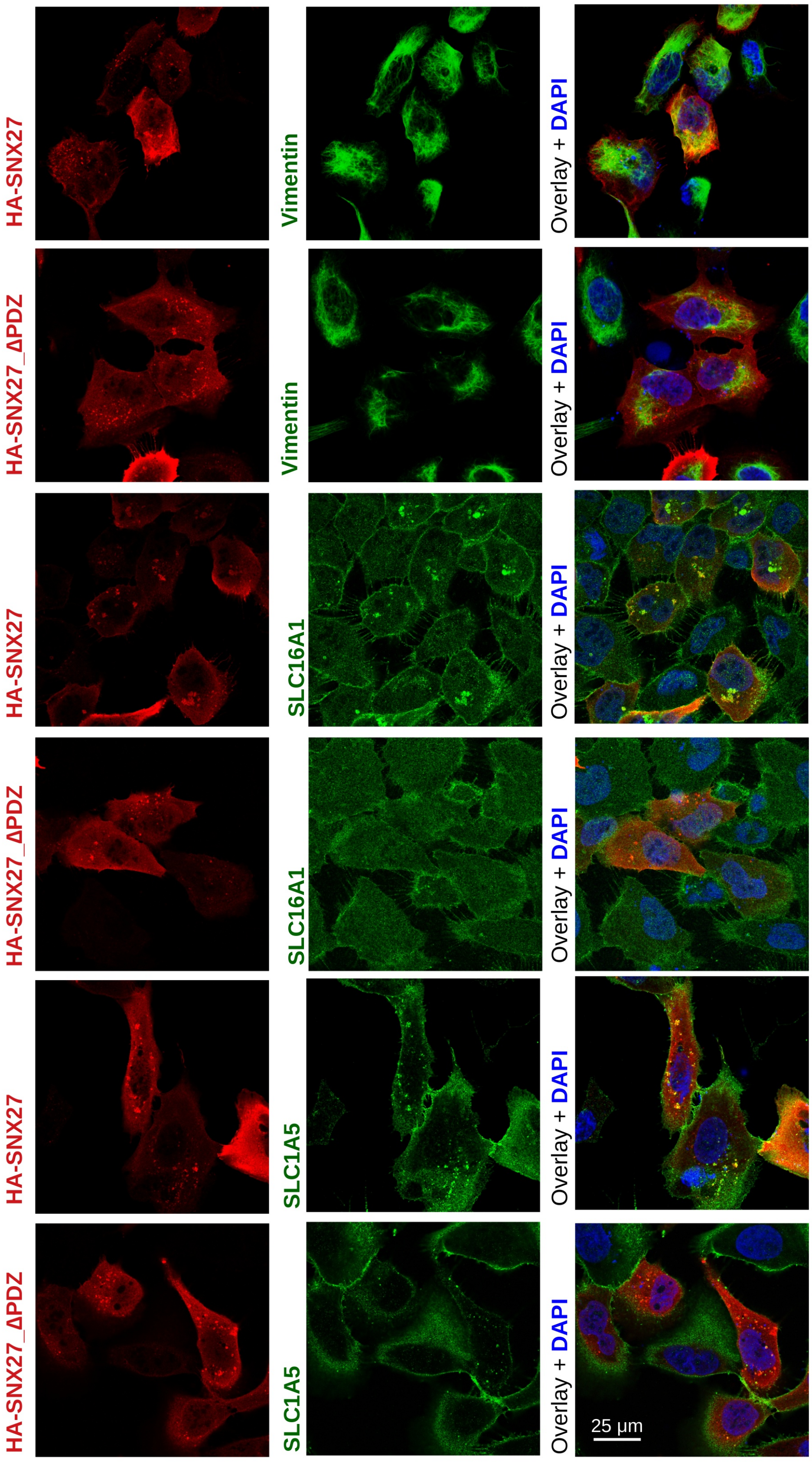

Supplementary Figure 7, Additional microscopic images for co-localization studies. Representative confocal images of U2OS cells expressing either HA-tagged SNX27 or HA-tagged SNX27_ΔPDZ. Cells were stained with antibodies against HA (red) and endogenous partner protein (Vimentin, SLC16A1, or SLC1A5, all in green), as well as with DAPI (blue).

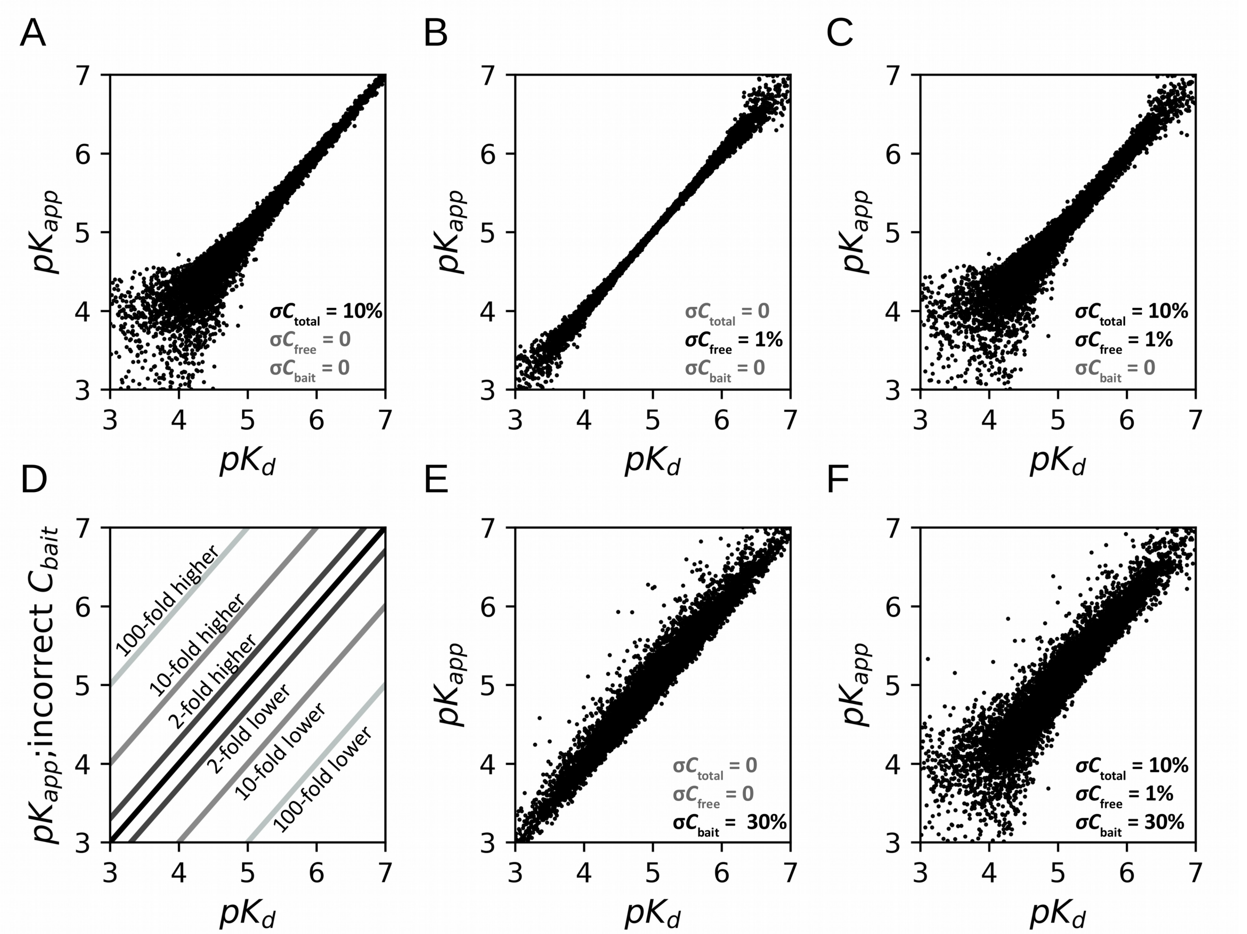

Supplementary Figure 8, Simulating error propagation in nHU assay. (A-C) A random error (with normal distribution) in either (A, B) or both (C) total of free concentration measurements affect the uncertainty of the determined BI value that propagates to the converted apparent affinities. (D) In case compared affinities were determined in a single experiment, the bait concentration does not have a standard deviation between the different affinities, but the estimated bait concentration can diverge from the true bait concentration, resulting in a constant drift in the observed affinities. (E, F) In case compared affinities were obtained from multiple nHU measurements, the uncertainty of the bait concentration between measurements also introduces error (E), that contributes to the overall error of each measurement caused by uncertainty of BI measurement (F). Artificially high standard deviations were chosen to visualize the additive effects of error propagation. In addition to these sources of error, many other factors may also contribute to the overall error in affinity estimation, such as variability in partial activities of proteins, various binding models, or incomplete binding (not reaching equilibrium with all partners). Most of these sources of errors can be identified and eliminated by using careful nHU titrations instead of single point measurements, where the error of affinity determination is mostly impacted by the inaccuracy of the bait concentration estimation.

Tables S1: Complementary source data for nHU experiments.

- Sheet 1: Detailed legends
- Sheet 2-3: nHU-WB experiments
- Sheet 4-12: nHU-MS experiments
- Sheet 13: Summary of affinities obtained from nHU-MS experiments.
